# Genome Sequences of vB_RleM_RL38JI and vB_RleM_RL2RES, Two Virulent Rhizobium leguminosarum Transducing Phages

**DOI:** 10.1128/MRA.01589-19

**Published:** 2020-03-12

**Authors:** K. M. Damitha Gunathilake, Supriya V. Bhat, Christopher K. Yost, Michael F. Hynes

**Affiliations:** a Biological Sciences, University of Calgary, Calgary, Canada; b Department of Biology, University of Regina, Regina, Canada; Loyola University Chicago

## Abstract

Phages vB_RleM_RL38JI and vB_RleM_RL2RES are known to mediate generalized transduction in Rhizobium leguminosarum. The RL38JI genome consists of 158,577 nucleotides and 270 predicted genes, whereas RL2RES has a 156,878-bp genome with 262 predicted genes. The two genomes are similar, with 82.88% nucleotide identity to each other.

## ANNOUNCEMENT

*Rhizobium* phages vB_RleM_RL38JI and vB_RleM_RL2RES are virulent and known for their ability to mediate generalized transduction in Rhizobium leguminosarum. Phage RL38JI was isolated from a pea field in Norfolk, United Kingdom (1). It was described as belonging to the family *Myoviridae* (2). The deoxycytidine residues in its genome were shown to be replaced with three different modified residues (3). RL2RES was isolated from soil collected from a field trial in Hertfordshire, United Kingdom (4). The morphology of RL2RES was described as being comparable to RL38JI, and both viruses belong to the family *Myoviridae* (4). RL38JI was provided to us for transduction experiments in the 1980s by John Beringer and Andrew Johnston. RL2RES was sent to us by Penelope Hirsch in 2003.

Phage lysates, using R. leguminosarum strain VF39SM as a bacterial host, were prepared using the modified method described by Halmillawewa et al. (5), based on previously described methods (4, 6); these protocols included a DNase treatment. The genomic DNA of the phages was purified using the method of Lech et al. (7), with the exception that a phage genome extraction kit (Norgen Biotek Corp., Canada) was used instead of phenol-chloroform extraction to extract DNA. The library preparation for sequencing was achieved with a Nextera XT index kit v2 (Illumina, Inc., USA). For each genome, paired-end reads of a 150-bp read length were generated using Illumina MiSeq instrumentation. Read qualities were evaluated using FastQC (8). Reads with a Phred quality score below 30 were removed using Trimmomatic v0.39 (9). The trimmed reads were assembled by Geneious-R11 v11.1 using default parameters. Assemblies gave a single contig for both phages.

The assembled genomes were annotated using Center for Phage Technology (CPT) Apollo available at the CPT Galaxy platform (https://cpt.tamu.edu/galaxy-pub/). Putative genes were predicted using PAP structural annotation workflow 2019 (v8.16), which integrates Glimmer (Galaxy v0.2) (10), MetaGeneAnnotator (Galaxy v1.0.0) (11), and ARAGORN for tRNA identification (Galaxy v19.1.0.0) (12). Possible functions of putative genes were predicted using PAP functional annotation workflow 2019 (v8.16), which includes database searches for conserved domains with InterProScan (Galaxy v5.33-72.0) (13) and for sequence similarities with BLAST+blastp (Galaxy v0.1.01) (14) at a 0.001 maximum expectation value. The search databases included the NCBI nonredundant database (15) and UniProtKB, Swiss-Prot, and TrEMBL (16).

The phylogenetic relationships were analyzed using CLUSTAL W 2.1 (17). PhageTerm (Galaxy v1.0.12) (18) was used to predict the phage termini and packaging mechanisms. The average nucleotide identity (ANI) of genomes by pairwise comparison was determined using JSpeciesWS (19; http://jspecies.ribohost.com/jspeciesws/). The amino acid sequence similarities of certain proteins were determined using NCBI+blastp (14) and EMBOSS Needle-protein (20; https://www.ebi.ac.uk/Tools/psa/emboss_needle/).

The two genomes of RL38JI and RL2RES are similar to each other, with an average nucleotide identity of 82.88%. The main features of these two genomes are indicated in Table 1. Both genomes are similar to previously reported T4-like rhizobiophage P10VF (GenBank accession number NC_025429.1), with about 88% ANI. The amino acid sequences of their terminase large subunits are 99.5% similar, while the major capsid proteins are identical.

**TABLE 1:** Main features of RL38JI and RL2RES phage genomes

Feature^*a*^	Value for phage:	
	RL38JI	RL2RES
Total no. of paired-end reads	34,828	40,310
Genome coverage (×)	27.342	37.737
Genome size (bp)	158,577	156,878
G+C content (%)	49.8	50
No. of predicted genes	270	262
No. of genes with predicted functions	61	60

a No tRNAs were identified in either phage.

Phylogenetic analysis with amino acid sequences of the terminase large subunit and major capsid protein placed them in the same cluster as P10VF and *Enterobacteria* phage T4. According to PhageTerm analysis, they have circularly permuted genomes with redundant ends and a headful genome packaging mechanism resembling that of phage T4.

### Data availability.

These genome sequences were deposited under GenBank accession numbers MN549360 (RL38JI) and MN549361 (RL2RES). The raw sequence reads are available at accession numbers SAMN13697382 (RL38JI) and SAMN13697383 (RL2RES), and both are under BioProject accession number PRJNA598046.
